# Frequency and machine learning predictors of severe depressive symptoms and suicidal ideation among university students

**DOI:** 10.1017/S2045796023000550

**Published:** 2023-07-07

**Authors:** Nicola Meda, Susanna Pardini, Paolo Rigobello, Francesco Visioli, Caterina Novara

**Affiliations:** 1 Department of Neuroscience, University of Padova, Padova, Italy; 2 Department of General Psychology, University of Padova, Padova, Italy; 3 Department of Molecular Medicine, University of Padova, Padova, Italy; 4 IMDEA-Food, CEI UAM + CSIC, Madrid, Spain

**Keywords:** college students, depression, prospective study, random forest, suicidality

## Abstract

**Aims:**

Prospective studies on the mental health of university students highlighted a major concern. Specifically, young adults in academia are affected by markedly worse mental health status than their peers or adults in other vocations. This situation predisposes to exacerbated disability-adjusted life-years.

**Methods:**

We enroled 1,388 students at the baseline, 557 of whom completed follow-up after 6 months, incorporating their demographic information and self-report questionnaires on depressive, anxiety and obsessive–compulsive symptoms. We applied multiple regression modelling to determine associations – at baseline – between demographic factors and self-reported mental health measures and supervised machine learning algorithms to predict the risk of poorer mental health at follow-up, by leveraging the demographic and clinical information collected at baseline.

**Results:**

Approximately one out of five students reported severe depressive symptoms and/or suicidal ideation. An association of economic worry with depression was evidenced both at baseline (when high-frequency worry odds ratio = 3.11 [1.88–5.15]) and during follow-up. The random forest algorithm exhibited high accuracy in predicting the students who maintained well-being (balanced accuracy = 0.85) or absence of suicidal ideation but low accuracy for those whose symptoms worsened (balanced accuracy = 0.49). The most important features used for prediction were the cognitive and somatic symptoms of depression. However, while the negative predictive value of worsened symptoms after 6 months of enrolment was 0.89, the positive predictive value is basically null.

**Conclusions:**

Students’ severe mental health problems reached worrying levels, and demographic factors were poor predictors of mental health outcomes. Further research including people with lived experience will be crucial to better assess students’ mental health needs and improve the predictive outcome for those most at risk of worsening symptoms.

## Introduction

Students’ mental health has attracted much more research after COVID-19-related lockdowns than ever before (Arsandaux *et al.*, 2021; Bennett *et al.*, 2022; Gestsdottir *et al.*, 2021; Hernández-Torrano *et al.*, 2020; Nuñez *et al.*, 2022; Robinson *et al.*, 2022; Villani *et al.*, 2021; Voltmer *et al.*, 2021; Yamamoto *et al.*, 2022). Prospective studies showed that young adults (generally defined as people aged between 18 and 25 years) enroled in university were affected by symptoms of poorer mental health than other working peers or adults well before restrictive measures were implemented (Blanco *et al.*, 2008; Granieri *et al.*, 2021); in fact, as previously reported, higher education is a risk factor for depressive symptoms, anxiety symptoms and suicidal thoughts (Karyotaki *et al.*, 2020). Furthermore, young adulthood is also a crucial transition phase, accompanied by the pursuit of self-efficacy and economic independence, higher education and new social needs. The Global Burden of Disease Study also evidenced that disability-adjusted life-years are at their highest in young adulthood (GBD 2019 Mental Disorders Collaborators, 2022), in line with epidemiological findings indicating this period of life as the typical age of onset of most mental disorders (Solmi *et al.*, 2022). To date, research on student mental health found that university support services, when in place, probably had little impact on the well-being of the general student population (Barnett *et al.*, 2021; Eisenberg *et al.*, 2011) due to various reasons, namely, low rates of the number of counsellors per student (generating long waitlists – (Blanco *et al.*, 2008; Cohen *et al.*, 2022; Lueck and Poe, 2021)), high levels of personal stigma hindering help-seeking behaviours (Eisenberg *et al.*, 2009) and few systemic interventions to address the determinants of (poor) mental health and prompt an early intervention to assess and treat the symptoms before they develop into a full-blown mental disorder (Upsher *et al.*, 2022) or treat effectively the students with chronic symptoms of mental disorders (Zivin *et al.*, 2009).

Although essential to tailor population-specific psychological interventions (Barnett *et al.*, 2021), little prospective evidence has been published regarding the prediction of mental health problems in university students (Ebert *et al.*, 2019; Suldo *et al.*, 2011; Tyssen *et al.*, 2008). A thorough assessment of student population needs and their psychological symptoms (Kitzrow, 2009) – leading to faster and more reliable diagnosis work-ups – is the first step for a pragmatic approach to design and evaluate the efficacy of specific mental health interventions. However, some studies investigated students’ mental health through variables strictly related to generalized anxiety disorder and depressive disorders, with little indulging in assessing demographic or other determinants of psychological well-being (Sheldon *et al.*, 2021).

To provide better services, prospective studies that evaluate risk factors at baseline (ideally at university/college enrolment) would be helpful to predict those students expected to need help in the short, medium and long term. Several contributions regarding the role of the COVID-19 pandemic evidenced (Meda *et al.*, 2021; Nomura *et al.*, 2022; Weber *et al.*, 2022) that such studies can be (i) conducted with little or no specific funding and (ii) they can help better understand the mental disorder development trajectory in a population at high risk of distress and maladjustment.

The present work is part of a larger prospective study named ECOS (vide infra) that evaluates the depressive, anxiety, eating and obsessive–compulsive symptoms of students and their determinants. Here, we report prospective data collected at enrolment and after 6 months of hundreds of Italian university students who completed several questionnaires investigating mental health problems. The objectives of this study were to establish the percentage of students suffering from severe symptoms common to depression, anxiety, obsessive–compulsive disorder and suicidal ideation at baseline; to elucidate the sociodemographic variables (e.g., gender, financial situation and field of study (Berger *et al.*, 2015; Volpe *et al.*, 2019)) associated with poor mental health at baseline and to evaluate the risk factors (e.g., depressive symptoms at enrolment) that dent the chances of symptoms improvement or otherwise make symptom aggravation probable. Moreover, we wanted to establish which self-report scale could explain a substantial part of current and future mental health problems. To do so, given the *a priori* knowledge of factors associated – mainly identified through cross-sectional studies – with worse mental health outcomes in students (Limone and Toto, 2022), we first employed binomial regression modelling to evince the association between demographic-individual factors and severe depressive, anxiety, obsessive–compulsive symptoms and suicidal ideation. Lastly, given the paucity of studies providing evidence for which risk factors are associated with future poorer mental health, we opted to adopt a supervised machine learning approach to automatically determine which factors at baseline could be leveraged to predict a change in symptoms (new onset, stability, improvement or worsening) after 6 months.

## Materials and methods

### Study cohort

All procedures described in this research were approved by the University of Padova Psychology Ethical Committee (Area 17 – ECOS: Eating, Compulsive and Obsessive Symptoms in Young Adults Protocol Ref. 3005) under the latest version of the Declaration of Helsinki. A significant part of the procedures described herein was also employed in two previous articles derived from the same protocol (Meda *et al.*, 2021; Novara *et al.*, 2022). Participants provided written informed consent to the study. Recruitment took place in Padova, Italy, between October 2019 and October 2020. From October 2019 to March 2020, at the start of different teaching classes, we presented the objectives of the prospective study in person, and the attending students were given a card with an URL. By accessing the URL, students could provide their informed consent and participate in the study.

From March 2020 onward, due to COVID-related restrictions, the study was described via prerecorded videos or remote presentations at the beginning of teaching classes. Throughout the slide show, students had the possibility to copy the URL redirecting to the informed consent. Enrolment and follow-up continued throughout 2022. After obtaining informed consent, participants were required to complete a demographic schedule, as well as self-reported mental health questionnaires on the REDCap web application (Harris *et al.*, 2009). Every 6 months since enrolment, the participants were automatically contacted by email and asked to participate in another data collection (for a total of six contacts and possible evaluations). That email contained a unique and personal e-address for each participant to access the platform and complete the questionnaires another time. With that unique link, the anonymous identity of the participant could be tracked longitudinally. A total of 1,902 students agreed to participate in the study (21.3% response rate, approximately 9,000 students were invited to participate) in the first cross-sectional study. Of this pool, 1,388 participants matched the target population characteristics (Italian native-speaker students aged 18–30 years); participants were excluded if they were non-native speakers, were outside the previous age range, or completed the questionnaires only in part. No other inclusion or exclusion criteria were applied. Students who participated in the study are (or were) enroled in Medicine and Surgery, Psychology/Mental Health and Neuroscience, Pharmacy and other health sciences, STEM sciences, Arts and Humanities and Law, Economics and Political Sciences.

At the second cross-sectional study (6 months after the first questionnaire administration), of the students (*N* = 1388) who completed the first wave of questionnaires, 768 students agreed to participate in the study a second time. Of these students, 557 were deemed eligible according to the previously outlined criteria. Sample characteristics at first cross-sectional and comparison between the subsample of dropouts to second-time participants are reported in Tables 1 and 2, respectively.

**Table 1.** Sample characteristic at baseline

	Females	Males	*p*
Sample size	992	396	
Age, mean (SD)	20.95 (2.01)	21.18 (2.17)	NS
Marital status, *n* (%)			
Not married	964 (97.2)	385 (97.2)	NS
Married	28 (2.8)	11 (2.8)	
BMI, *n* (%)			
Normal weight	832 (83.9)	317 (80.1)	<0.001
Obesity	13 (1.3)	5 (1.3)	
Overweight	66 (6.7)	63 (15.9)	
Underweight	81 (8.2)	11 (2.8)	
Any mental disorder, *n* (%)			
Disorder	178 (17.9)	41 (10.4)	NS
No disorder	814 (82.1)	355 (89.6)	
Family history of MD, *n* (%)			
No	697 (70.3)	309 (78.0)	NS
Yes	295 (29.7)	87 (22.0)	
Major in, *n* (%)			
Arts and Humanities	86 (8.7)	15 (3.8)	<0.001
Law, Economics and Political Sciences	89 (9.0)	32 (8.1)	
Medicine and Surgery	329 (33.2)	136 (34.3)	
Pharmacy and Other HS	140 (14.1)	25 (6.3)	
Psychology/Mental Health, Neuroscience	111 (11.2)	14 (3.5)	
STEM	237 (23.9)	174 (43.9)	
Enrolment with respect to first lockdown, *n* (%)			
Before	592 (59.7)	224 (56.6)	<0.001
During	174 (17.5)	33 (8.3)	
After	226 (22.8)	139 (35.1)	
Any physical disorder, *n* (%)			
No	823 (83.0)	361 (91.2)	<0.001
Yes	169 (17.0)	35 (8.8)	
Any eating disorder, *n* %)			
No	924 (93.1)	396 (100.0)	<0.001
Yes	68 (6.9)	0 (0.0)	
BDI-II score, median [IQR]	11.00 [6.00, 19.00]	9.00 [4.00, 16.00]	<0.001
BAI score, median [IQR]	13.00 [7.00, 22.00]	9.00 [4.00, 16.00]	<0.001
OCI-R score, median [IQR]	15.00 [8.00, 22.00]	14.00 [9.00, 23.00]	NS
Suicidal ideation, *n* (%)			
No	830 (83.7)	301 (76.0)	NS
Yes	162 (16.3)	95 (24.0)	
Depression severity, *n* (%)			
Nil-Mild	685 (69.1)	265 (66.9)	NS
Moderate	82 (8.3)	53 (13.4)	
Severe	225 (22.7)	78 (19.7)	
Anxiety severity, *n* (%)			
Nil	278 (28.0)	174 (43.9)	<0.001
Mild	403 (40.6)	119 (30.1)	
Moderate	141 (14.2)	66 (16.7)	
Severe	170 (17.1)	37 (9.3)	
OC severity, *n* (%)			
Nil-Mild	539 (54.3)	224 (56.6)	NS
Moderate	311 (31.4)	115 (29.0)	
Severe	142 (14.3)	57 (14.4)	
Psychiatric medication – Past, *n* (%)			
No	92 (51.1)	10 (23.8)	NS
Yes	88 (48.9)	32 (76.2)	
Psychiatric medication – Now, *n* (%)			
No	48 (54.5)	13 (40.6)	NS
Yes	40 (45.5)	19 (59.4)	
Psychotherapeutic treatment – Past, *n* (%)			
No	26 (14.7)	7 (16.7)	NS
Yes	151 (85.3)	35 (83.3)	
Psychotherapeutic treatment – Now, *n* (%)			
No	71 (47.0)	24 (68.6)	NS
Yes	80 (53.0)	11 (31.4)	

BAI = Beck Anxiety Inventory; BDI = Beck Depression Inventory; BMI = body mass index; MD = mental disorders; OC = obsessive–compulsive; OCI-R = Obsessive–Compulsive Inventory-Revised. Statistical significance was assessed with *t*-test (for data with normal distribution), Mann-Whitney *U* test (for data with non-normal distribution) or Chi-square test for frequency data. Test characteristics (i.e., degrees of freedom are not reported for ease of readability). Further testing corrected for possible confounders can be found in the results section.

**Table 2.** Regression model for severe depressive symptoms

	BDI severity
Predictors	Odds ratios
Intercept	0.21*** (0.12–0.36)
Economic worry: Low (Reference: None)	0.65 (0.40–1.08)
Economic worry: Moderate	1.26 (0.80–1.98)
Economic worry: High	3.11*** (1.88–5.15)
Economic worry: Extreme	3.51** (1.63–7.57)
Residence: Commute (Reference: in Padova)	0.57** (0.38–0.85)
Residence: Off-site	0.64* (0.42–0.98)
Enrolment time: Before lockdown (Reference: after lockdown)	0.50*** (0.35–0.70)
Enrolment time: During lockdown	0.75 (0.47–1.19)
Followed 1 or more diets lifetime: Yes (Reference: No)	1.77*** (1.31–2.39)
Suicidal ideation: Yes (Reference: No)	9.29*** (6.71–12.86)
Observations	1388
BIC	1214.7
Marginal *R*^2^/Conditional *R*^2^	0.310/NA

* *p* < 0.05; **p < 0.01; ***p < 0.001.

BIC = Bayesian Information Criterion; BDI = Beck Depression Inventory-II.

### Measures

Participants were asked to report their demographic information, years of education, enrolment status, if they have ever been diagnosed with a mental health condition or other medical disorder, illness or disease or if they had a family history of mental health issues. Further questions investigated lifestyle habits, such as dietetic regimen, medication prescription, past/current psychotherapy and frequency of worry about their economic/financial conditions. The latter information was assessed with a 5-point Likert scale, where 0 = never worry (nil); 1 = rarely worried (mild); 2 = sometimes worried (moderate); 3 = frequently worried (high); 4 = always worried (extreme). To specifically design this measure, we defined the item similarly to the Worry Domains Questionnaire (Tallis *et al.*, 1992). All demographic variables were treated as categorical.

Five self-report questionnaires were administered: the Beck Depression Inventory-II (BDI-II; Beck *et al.*, 2011; Sica and Ghisi, 2007) to assess depression characteristics (in our sample, this measure maintained a high internal consistency, Cronbach’s *α* = 0.91 [range: 0.91–0.92]); the Beck Anxiety Inventory (BAI; Sica and Ghisi, 2007; Steer and Beck, 1997) for measuring the physical and cognitive symptoms of anxiety (Cronbach’s *α* = 0.92 [range: 0.91–0.93]); the Obsessive–Compulsive Inventory-Revised (OCI-R; Abramowitz and Deacon, 2006; Sica *et al.*, 2009); the Eating Disorder Inventory-3 (EDI-3; Garner, 2004; Giannini *et al.*, 2008 and the Eating Habits Questionnaire (EHQ; Gleaves *et al.*, 2013; Novara *et al.*, 2017) [52–71]. For ease of readability and consistency, in this article, we will discuss only the results related to depressive, anxious and obsessive symptoms, as well as suicidal ideation (the latter measured by item 9 of the BDI-II (Desseilles *et al.*, 2012; Green *et al.*, 2015)).

### Statistical analysis

Anonymized data were downloaded from the REDCap platform and curated using RStudio-R version 4.1.2 (R Core Team (2019)). In this work, we investigated the variables associated with depressive, anxious and obsessive–compulsive symptoms and suicidal ideation at baseline, as well as baseline predictors of mental health outcomes at the first follow-up (6 months after baseline). We analyse factors associated with more severe symptoms of depression, anxiety and obsessive–compulsive behaviour. For the purpose of binomial regression analysis and using random forest algorithms, we labelled participants with severe symptomatology (=1) or without it (=0), as measured with self-report measures. The reasons for employing a stringent cut-off are twofold: first, putative cut-offs for distinguishing nil, mild and moderate severity of symptoms are less reliable; second, the number of possible changes (and stability) in the severity is four times the number of possible changes with a single cut-off (see also below in the ‘Prospective assessment’ section). The cut-offs applied were based on the validation procedure of the single psychometric tools. They are as follows: 95th percentile of the BDI-II score (i.e., female score above 20 and male score above 19); score above 26 for the BAI scale (derived from the validation study, for both genders) and score above 28 for the OCI-R scale (derived from the validation study based on the AUC, for both genders). Suicidal ideation was measured with item 9 of the BDI scale (Desseilles *et al.*, 2012; Green *et al.*, 2015), with any score above 0 meaning the presence of some kind of suicidal ideation. The rationale for this choice is supported by literature, which shows little advantage in distinguishing between active or passive suicidal ideation when assessing risk (Liu *et al.*, 2020).

First, we implemented a regression modelling approach to evaluate the weight of several independent variables on the outcomes of interest, while covarying for possible confounders. In this case, we used a binomial distribution to model the data and show the association between severe symptoms and independent variables. Here we report only the models with the lowest Bayesian Information Criterion (BIC – an index of model fitting: the lower the BIC, the lower the variance left unexplained by the model, the better the fit), which were identified through a stepwise selection approach (Raftery, 1995). We report the β estimate for each variable of the model with the lowest BIC. The estimation of a variable represents the importance of that variable in changing the questionnaire scores.

### Prospective assessment

Then, to identify the predictors of severity changes from baseline to 6 months after enrolment, we implemented a random forest algorithm (supervised machine learning), which extracted from the dataset the independent variables at baseline that played a statistically significant role in determining the change or stability of the severity of the symptoms (i.e., stable ‘well-being’ – not severe at baseline and after 6 months; stable ‘severity’ – severe both at baseline and after 6 months; improvement – from severe to not severe symptoms in 6 months; worsening – from not severe to severe in 6 months). In this way, the machine learning algorithm was asked to predict four different outcomes. The algorithm was first trained using 80% of the dataset and then tested to assess its performance on the remaining 20% of the dataset. Random forest explainer package (Paluszynska *et al.*, 2020) aided the visualization of random forest characteristics and predictive performance.

## Results

A total of 1,388 students participated in the study (992 – 71.4% females; aged 21.01 ± 2.05). Some relevant statistically significant differences between the genders emerged with an uncorrected direct comparison approach (Table 1). Females were more likely to be underweight (8.2%) than males (2.8%), who, on the other hand, reported being overweight more frequently (15.19%) than females (6.7%). Most participants were recruited before the first COVID-19-related lockdown in Italy (which lasted from the second week of March 2020 to the first week of May 2020).

Females were also more likely to suffer from any physical disorder (17%) or eating disorder (6.9%) than males (8.8% and 0%, respectively). Moreover, median scores for depressive and anxiety symptomatology were higher in females (11, interquartile range [6–19] and 13, IQR [7–22], respectively) than males (9, IQR [4–16] for both measures). However, the frequency of severe depression symptoms was not different between sexes: only the frequency of severe anxiety symptoms was higher in females than in males. It is noteworthy that the frequency of suicidal ideation was not different between the sexes.

Importantly, there were no statistically significant differences in the frequency of self-reported, clinician-diagnosed mental health condition diagnosis between sexes or family history of mental disorders.

**Figure 1.**
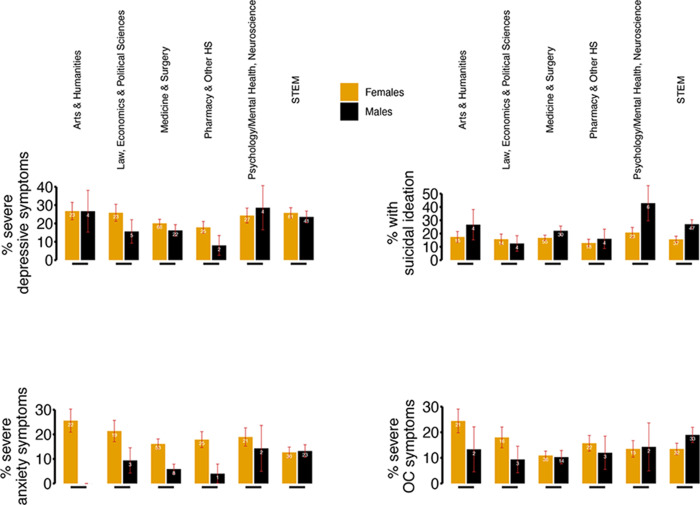
Frequency of severe depressive, anxiety symptoms and suicidal ideation among students per field of study and gender.

For a quick comparison (uncorrected for confounder variables), we reported in Figure 1 the percentage of severe symptoms (of depression, anxiety or obsessive–compulsive symptomatology), as well as the frequency of suicidal ideation per gender and field of study.

OC = obsessive–compulsive (symptoms); HS = Health Sciences; STEM = Science, Technology, Engineering and Mathematics. Orange bars represent female participants and black bars male participants. In red, inside each bar: error bar; the number on top of the orange/black bar represents the absolute number (white) of participants in a specific field of study experiencing severe symptoms. Absolute numbers and frequencies are also detailed in Table 1.

### Significant variables associated with severe symptoms of depression

We define several binomial regression models to assess the contribution of demographic variables and clinically relevant characteristics to the higher severity of depression, anxiety, obsessive–compulsive symptoms and the presence of suicidal ideation.

The model that best described the severity of depressive symptoms, as measured with the Beck Depression Inventory, is reported in Table 2. We evidenced an association between high and extreme frequency of worry for one’s economic situation, with the odds of having severe symptoms of depression approximately tripled (odds ratio [OR] for high frequency of worry: 3.11 [1.88–5.15], *p* < 0.001; OR for extreme frequency of worry: 3.51 [1.53–7.57], *p* < 0.01). Additionally, severe depressive symptoms were associated with having followed at least one diet during life (probability of severe depressive symptoms increased by approximately 70%; OR = 1.77 [1.31–2.39], *p* < 0.001). The largest association was between suicidal ideation and depression severity, with the former increasing the odds of depression severity by approximately nine times (OR = 9.29 [6.71–12.86], *p* < 0.001). We also evinced some factors associated with a lower likelihood of manifesting severe symptoms: either commuting or living off-site (residence outside of Padova by more than 50 km), as well as whether enrolment in the study took place before the first ever COVID-19-related lockdown (i.e., the restrictive measures put in place from March to May 2020). Both variables approximately halved the chances of participants reporting severe symptoms of depression. The performance of the model was decent, with *R*^2^ = 0.310.

### Significant variables associated with severe anxiety symptoms and obsessive–compulsive symptoms

For each of the other measures (i.e., anxiety severity, obsessive–compulsive symptoms severity and presence of suicidal ideation), we reported two models: one agnostic with respect to the BDI-II total score and its subscale score, the other instead leveraged this information to produce a better fit of the data to the model. The reason for describing these two models (i.e., with or without the information produced by the BDI-II) is that the BAI, OCI-R scores, and the presence of suicidal ideation (measured with item number nine of the BDI-II) might be influenced by the presence of depression/depressive symptoms (i.e., the higher the burden of depressive symptoms, the higher the likelihood of these scores being elevated).

We found one variable associated with severe symptoms of anxiety: suicidal ideation. Its presence increased twofold (Table 3A, OR = 2.09 [1.49–2.93], *p* < 0.001, Table 2) the odds of severe symptoms. Using the score of BDI-II and its subscales, the previous association was no longer significant and replaced by the somatic subscale score of BDI-II (OR = 1.09 [1.07–1.12], *p* < 0.001 – meaning that, for each point on the scale, the odds of severe anxiety symptoms increased by approximately 9%). However, for both models, performance was very low (*R*^2^ = 0.034 or 0.07, respectively).

**Table 3.** Regression models for severe anxiety or obsessive–compulsive symptoms

	A		B	
	BAI severity	BAI severity	OCI-R severity	OCI-R severity
Predictors	Odds ratios	Odds ratios	Odds ratios	Odds ratios
Intercept	0.18*** (0.14–0.24)	0.08*** (0.06–0.11)	0.12*** (0.07–0.19)	0.04*** (0.03–0.05)
Enrolment time: Before lockdown (Reference: After lockdown)	0.80 (0.57–1.11)		0.55*** (0.39–0.77)	
Enrolment time: During lockdown	0.60 (0.36–1.00)		0.84 (0.52–1.34)	
Suicidal ideation: Yes (Reference: No)	2.09*** (1.49–2.93)		2.41*** (1.71–3.42)	
Economic worry: Low (Reference: None)			1.04 (0.59–1.82)	
Economic worry: Moderate			1.97** (1.18–3.30)	
Economic worry: High			3.08*** (1.75–5.42)	
Economic worry: Extreme			2.10 (0.91–4.85)	
BDI – Somatic subscale		1.09*** (1.07–1.12)		
BDI – Total score				1.10*** (1.08–1.11)
Observations	1388	1388	1388	1388
BIC	1182.2	1138.4	1131.6	995.0
Marginal *R*^2^	0.034	0.071	0.113	0.206

* *p* < 0.05; **p < 0.01; ***p < 0.001.

BIC = Bayesian Information Criterion; BAI = Beck Anxiety Inventory; BDI = Beck Depression Inventory-II; OCI-R = Obsessive–Compulsive Inventory-Revised.

Regarding obsessive–compulsive symptoms, we found that the frequency of economic worry increased by two to three times the odds of severe symptoms (Table 3B, moderate frequency OR = 1.97 [1.18–3.30], *p* < 0.01; high frequency OR = 3.08 [1.75–5.42], *p* < 0.001; extreme frequency was not significant in this model); furthermore, enrolment before lockdown decreased the odds of severe symptoms by 50% (OR = 0.55 [0.39–0.77], *p* < 0.001). Finally, suicidal ideation increased the probability of severe symptoms by two times and a half – OR of 2.41 ([1.71–3.42], *p* < 0.001). The model fit was low (*R*^2^ = 0.113). However, considering the BDI-II total score in the model provided a better fit (although still low, *R*^2^ = 0.2) for each additional point on the scale, the odds of having severe symptoms increased by 10% ([1.08–1.11], *p* < 0.001).

### Significant variables associated with current suicidal ideation

A large number of variables were significantly associated with suicidal ideation: we highlighted a protective role of enrolment before lockdown (Table 4, OR = 0.56 [0.4–0.78], *p* < 0.001; meaning that before lockdowns, the risk of suicidal ideation was halved with respect to after restrictive measures were effective) and marginally significant protection linked to enrolment during lockdown (OR = 0.58, [0.36–0.95], *p* < 0.05). Having no history of diagnosed mental disorder was found to be a significant protective factor with a large effect (OR = 0.28 [0.2–0.4], *p* < 0.001; i.e., reducing the chances of suicidal ideation by approximately 4 times).

**Table 4.** Regression models for current suicidal ideation

	Current suicidal ideation	Current suicidal ideation
Predictors	Odds ratios	Odds ratios
Intercept	0.33*** (0.18–0.58)	0.03*** (0.02–0.05)
Gender: Male (Reference: female)	2.18*** (1.57–3.01)	2.84*** (1.87–4.32)
Economic worry: Low (Reference: None)	1.10 (0.66–1.84)	
Economic worry: Moderate	2.27*** (1.41–3.64)	
Economic worry: High	2.60*** (1.52–4.45)	
Economic worry: Extreme	10.42*** (5.04–21.52)	
Family history of mental disorder(s): Yes (Reference: No)	1.60** (1.17–2.20)	1.48* (1.00–2.18)
Enrolment time: Before lockdown (Reference: After lockdown)	0.56*** (0.40–0.78)	
Enrolment time: During lockdown	0.58* (0.36–0.95)	
BMI: Obese (Reference: Normal weight)	2.93* (1.01–8.50)	
BMI: Overweight	0.99 (0.62–1.60)	
BMI: Underweight	1.14 (0.64–2.04)	
History of mental disorder(s): No (Reference: Yes)	0.28*** (0.20–0.40)	0.58* (0.38–0.9)
BDI – Cognitive subscale		1.36*** (1.31–1.42)
Observations	1388	1388
BIC	1264.0	1114
Marginal *R*^2^/Conditional *R*^2^	0.195	0.463

* *p* < 0.05; **p < 0.01; ***p < 0.001.

BIC = Bayesian Information Criterion; BDI = Beck Depression Inventory-II; BMI = body mass index.

Regarding risk factors, male gender increased the odds of suicidal ideation approximately twofold (OR = 2.18 [1.57–3.01], *p* < 0.001). Also, the frequency of economic worry was identified as a possible risk factor, with increasing odds of suicidal ideation, as frequency varied from moderate to extreme (for the latter, a 10-fold increase was evidenced [5–21.5], *p* < 0.001). A family history of mental disorder was associated with 60% higher odds ([1.17–2.2], *p* < 0.01) of suicidal ideation. A statistically marginal significance of BMI was shown (being obese increased the odds of suicidal ideation about three times, OR = 2.93 [1.01–8.5], *p* < 0.05).

When covarying for BDI-II scores, we found that the score to the cognitive subscale of depression (which measured cognitive distortions) increased the odds of suicidal ideation by 36% for each additional point to the subscale ([1.31–1.42], *p* < 0.001) – in this case, for the scoring of the cognitive subscale, we excluded item 9 pertaining to suicidal ideation due to collinearity with the outcome measure. The absence of a history of mental disorders maintained a significant protective role, although the size of the effect was reduced (OR = 0.58 [0.38–0.9], *p* < 0.05). However, a marginal significance was achieved for the family history of mental disorder (if present, the risk of suicidal ideation increased by 48%; OR = 1.48 [1.00–2.18], *p* < 0.05). It should be noted that the size of the effect of the male sex increased, with the odds of suicidal ideation in men being 2.84 times higher than in women ([1.87–4.32], *p* < 0.001). The *R*^2^ for this model was 0.46 and should be considered highly acceptable (considering the volatility of the outcome measure).

### Comparison between follow-up and drop-out samples

Six months after enrolment, 557 participants completed a second wave of questionnaires (Table 5). We compared the samples of participants who participated in follow-up or dropped out. Follow-up completers were significantly more likely to have a family history of mental disorders, study Medicine and Surgery and be enroled before lockdown. No other statistically significant variables were evidenced.

**Table 5.** Comparison between follow-up completers and dropouts

	Follow-up completers	Dropouts	*p*
Sample size	557	814	
Age, mean (SD)	21.10 (2.09)	20.96 (2.05)	NS
Gender, *n* (%)			
Females	424 (76.1)	557 (68.4)	NS
Males	133 (23.9)	257 (31.6)	
Marital status, *n* (%)			NS
Not married	541 (97.1)	792 (97.3)	
Married	16 (2.9)	22 (2.7)	
BMI, *n* (%)			
Normal weight	461 (82.8)	676 (83.0)	NS
Obesity	7 (1.3)	11 (1.4)	
Overweight	46 (8.3)	80 (9.8)	
Underweight	43 (7.7)	47 (5.8)	
Any mental disorder, *n* (%)			
Disorder	106 (19.0)	110 (13.5)	NS
No disorder	451 (81.0)	704 (86.5)	
Family history of MD, *n* (%)			
No	374 (67.1)	619 (76.0)	<0.001
Yes	183 (32.9)	195 (24.0)	
Major in, *n* (%)			
Arts and Humanities	45 (8.1)	54 (6.6)	<0.001
Law, Economics and Political Sciences	33 (5.9)	86 (10.6)	
Medicine and Surgery	241 (43.3)	218 (26.8)	
Pharmacy and Other HS	37 (6.6)	126 (15.5)	
Psychology/Mental Health, Neuroscience	57 (10.2)	67 (8.2)	
STEM	144 (25.9)	263 (32.3)	
Enrolment with respect to first lockdown, *n* (%)			
Before	363 (65.2)	442 (54.3)	<0.001
During	73 (13.1)	133 (16.3)	
After	121 (21.7)	239 (29.4)	
Any physical disorder, *n* (%)			
No	479 (86.0)	689 (84.6)	NS
Yes	78 (14.0)	125 (15.4)	
Any eating disorder, *n* (%)			
No	523 (93.9)	781 (95.9)	NS
Yes	34 (6.1)	33 (4.1)	
BDI-II score, median [IQR]	11.00 [6.00, 19.00]	10.00 [5.00, 17.00]	NS
BAI score, median [IQR]	13.00 [6.00, 22.00]	11.00 [6.00, 20.00]	NS
OCI-R score, median [IQR]	15.00 [8.00, 23.00]	14.00 [8.00, 22.00]	NS
Suicidal ideation, *n* (%)			
No	436 (78.3)	681 (83.7)	NS
Yes	121 (21.7)	133 (16.3)	
Depression severity, *n* (%)			
Nil-Mild	373 (67.0)	563 (69.2)	NS
Moderate	49 (8.8)	85 (10.4)	
Severe	135 (24.2)	166 (20.4)	
Anxiety severity, *n* (%)			
Nil	175 (31.4)	270 (33.2)	NS
Mild	211 (37.9)	305 (37.5)	
Moderate	81 (14.5)	123 (15.1)	
Severe	90 (16.2)	116 (14.3)	
OC severity, *n* (%)			
Nil-Mild	300 (53.9)	454 (55.8)	NS
Moderate	179 (32.1)	242 (29.7)	
Severe	78 (14.0)	118 (14.5)	
Psychiatric medication – Past, *n* (%)			
No	45 (42.1)	56 (50.0)	NS
Yes	62 (57.9)	56 (50.0)	
Psychiatric medication – Now, *n* (%)			
No	31 (50.0)	30 (53.6)	NS
Yes	31 (50.0)	26 (46.4)	
Psychotherapeutic treatment – Past, *n* (%)			
No	13 (12.4)	19 (17.1)	NS
Yes	92 (87.6)	92 (82.9)	
Psychotherapeutic treatment – Now, *n* (%)			
No	46 (50.0)	49 (53.3)	NS
Yes	46 (50.0)	43 (46.7)	

BAI = Beck Anxiety Inventory; BDI = Beck Depression Inventory; BMI = body mass index; MD = mental disorders; OC = Obsessive–Compulsive; OCI-R = Obsessive–Compulsive Inventory-Revised. Statistical significance was assessed with *t*-test (for data with normal distribution), Mann-Whitney *U* test (for data with non-normal distribution) or Chi-square test for frequency data. Test characteristics (i.e., degrees of freedom) are not reported for ease of readability.

### Prediction of severe depressive symptoms after 6 months with supervised machine learning

**Figure 2.**
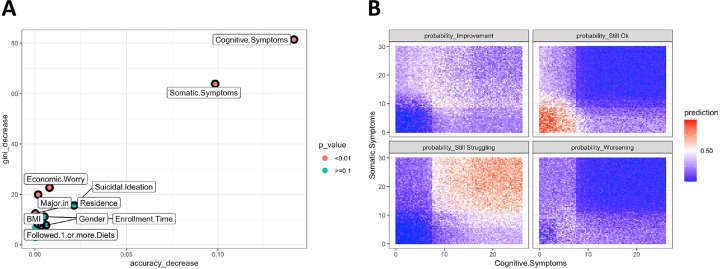
Significant variables extracted by random forest algorithms to predict severe depressive symptoms at follow-up. (A) Multiway plot showing the relative importance of the top 10 variables used to predict severe depressive symptoms at follow-up (6 months after enrolment). The variables in red are statistically significant. A higher score in accuracy decrease or gini decrease (which is a measure of how each variable contributes to the homogeneity of the nodes and leaves) reflects the relative importance of that variable in the model (i.e., if the variable is located in the upper right corner of the plot – like cognitive symptoms – it means that removing that variable from the model significantly worsens the model prediction capability). (B) Predictions of the random forest of severe depressive symptoms depending on values of the BDI-II subscale scores at baseline. Participants could be classified into four classes: improvement from baseline, worsening from baseline or stability (either steady severe symptoms or steady well-being). For each subplot predictions range from 0 (deep blue) to 1 (red). For example, the probability of ‘still struggling’ reaches almost 1 (certainty) if the subscale scores at the beginning of the study are high.

We implemented a random forest algorithm to determine which variables at baseline could be leveraged to predict a change (or stationarity) in depressive symptoms. The algorithm was trained to predict four possible outcomes: stationarity of severe symptoms, stationarity of well-being, improvement of symptoms or worsening symptoms: 79 participants experienced a worsening of symptoms – 45 from nil-mild symptoms to moderate symptoms or from moderate to severe; 34 from nil-mild symptoms to severe symptoms; 77 participants experienced a symptom improvement; 310 remained in a state of well-being, whereas 91 participants still experienced severe symptoms. The random forest trained with 80% of the dataset (*n* = 461) identified cognitive and somatic subscale scores of BDI-II, frequency of economic worry and field of study as significant variables to classify participants into the four classes (Figure 2A). Furthermore, the interaction between the two subscales (Figure 2B) showed that the predictive capacity of the algorithm to classify participants is heavily influenced by extreme scores: the chances of participants still struggling with severe symptoms after 6 months from enrolment reached a probability of almost 1 when both subscale scores were higher than 20; similarly, the probability of being without severe symptoms (subpanel ‘Still Ok’ of Figure 2A) reached almost 1 when both subscale scores are below 10. When tested in the remaining 20% (*n* = 96) of the sample, the overall precision of the model was 0.77 (95% CI: 0.67–0.85), which was statistically significant (*p* < 0.001), although with lower precision in predicting participants whose symptoms worsened (balanced precision: 0.49) than participants stationarily well (0.85). In particular, the positive predictive value of worsening symptoms after 6 months of enrolment was 0 (whereas the negative predictive value was 0.89). Further details on model characteristics can be found in the Supplementary Appendix.

### Prediction of suicidal ideation after 6 months with supervised machine learning

**Figure 3.**
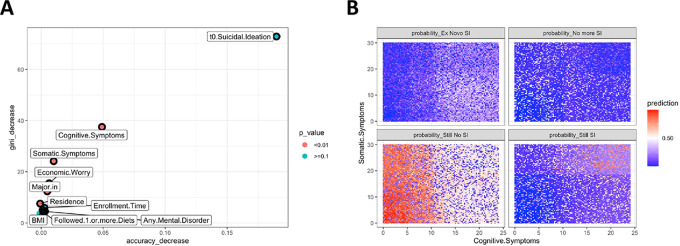
Significant variables extracted by random forest algorithms to predict suicidal ideation at follow-up. (A) Multiway plot showing the relative importance of the top 10 variables used to predict suicidal ideation at follow-up. The variables in red are statistically significant. A higher score in the decrease in accuracy or the decrease in gini reflects the relative importance of that variable in the model. (B) Predictions of the random forest of suicidal ideation according to the values of the BDI-II subscale scores at baseline.

A random forest algorithm was trained to predict four possible outcomes: stationarity of suicidal ideation, stationarity of no suicidal ideation and improvement (suicidal ideation at baseline, not at follow-up) or worsening symptoms (no suicidal ideation at baseline, present at follow-up – termed *ex novo*): 34 participants report *ex novo* suicidal ideation, while 48 participants reported absence of suicidal ideation at follow-up; 402 without suicidal ideation both at baseline and at follow-up; 73 reported suicidal ideation both at baseline and follow-up. The random forest trained with 80% of the data set (*n* = 461) identified cognitive and somatic subscale scores of BDI-II, frequency of economic worry, field of study and residence (in Padova, commuting or off-site), as significant variables to classify participants into the four classes (Figure 3A). Noteworthily, although suicidal ideation at baseline could be leveraged by the model to improve predictive capability, it was not statistically significant. Furthermore, the interaction between the two subscales of the BDI (Figure 3B) showed that the predictive capacity of the classification algorithm is again heavily influenced by extreme scores. The chances of participants not reporting suicidal ideation were high for cognitive scores below 10, with little contribution of somatic symptoms scores. The model classification performance was low for the other classes (improvement, worsening or suicidal ideation at baseline and follow-up). When tested in the remaining 20% (*n* = 96) of the sample, the overall precision of the model was 0.91 (95% CI: 0.84–0.96), which was statistically significant (*p* < 0.001). However, the balanced accuracy for *ex novo* suicidal ideation was 0.5 with a negative predictive value of 0.96 but a sensitivity of 0. Balanced accuracy for participants who were stationary – without suicidal ideation – was 0.92. Further details on model characteristics can be found in the Supplementary Appendix.

## Discussion

Since the COVID-19 pandemic and the resulting lockdowns, mental health among students has attracted more research than ever before (Li *et al.*, 2021; Robinson *et al.*, 2022). Several prospective studies before the pandemic highlighted that higher education is a risk factor for depressive symptoms, anxiety symptoms and suicidal thoughts (Adams *et al.*, 2021; Storrie *et al.*, 2010).

However, there is little prospective evidence on the prediction of mental health problems. This would be the first step in a pragmatic approach to predicting which students are likely to need assistance in the short term, medium term and long term.

In this work, we assessed students’ depressive, anxiety, obsessive–compulsive symptoms and suicidal ideation and their possible determinants. We collected demographic and self-report measures from 1,388 Italian university students who completed questionnaires on mental health problems at enrolment and after 6 months. We (i) report a high percentage of students suffering from severe symptoms of mental health problems; (ii) identify the factors associated with poor mental health at baseline (for depressive symptoms: economic worry, enrolment time with respect to lockdowns, having followed at least one diet and suicidal ideation; for suicidal ideation: male gender, family or own history of mental disorder(s) and cognitive depressive symptoms; for obsessive–compulsive and anxiety symptoms: severity of depressive symptoms severity); (iii) highlight the risk factors that could predict a lack of improvement in symptoms or an increased likelihood of symptom worsening (cognitive symptoms and somatic symptoms of depression, economic worry, field of study and residence for both severity of depressive symptoms and suicidal ideation). Lastly, we showed which self-report measures explained a significant part of data variance related to current and future mental health problems.

We found that the percentage of students suffering from severe depressive symptoms ranged between 22% (in females) and 17% in males. That means that in our sample, 1 out of 4–6 had scored higher than the 95th percentile of normative scores for the BDI-II (the questionnaire used to assess depressive symptoms). Another extremely distressing finding was that 22% and approximately 20% (males and females, respectively) endorsed suicidal ideation in the 2 weeks before questionnaire completion. Figures for severe anxiety and obsessive–compulsive symptoms are a tad lower (severe anxiety percentages are approximately between 9% and 17%; severe OC symptoms are 14%). These findings are generally in line with previous descriptive studies on the percentage of students with mental health problems (Robinson *et al.*, 2022; Sheldon *et al.*, 2021), although our results were, to some extent, more worrying than expected. We consider this finding secondary to the normalization of mental health problems after the wave of COVID-19 studies, which reported that an eye-watering percentage of young adults struggled with their mental health (before, during and after lockdowns) and on which an astounding amount of media reports were recorded. Future studies could test this ‘de-stigmatization’ hypothesis by measuring mental health problems and the degree of personal and social stigma in university students and compare it to historical data to assess if a sort of Papageno effect (Etzersdorfer and Sonneck, 1998; Niederkrotenthaler *et al.*, 2010) for general mental health symptoms could be in place (Papageno effect, strictly speaking, is ‘the [positive] influence that mass media can have by responsibly reporting on suicide and presenting non-suicide alternatives to crises’).

In our study, we found that people who reported high or extreme levels of worry about the economic situation were three times more likely to experience severe symptoms of depression compared to those who reported lower levels of worry. The effect between the economic situation and depressive symptoms is a bidirectional vicious cycle (Ridley *et al.*, 2020). The results of the random forest algorithm we employed also evidenced this link, as it identified economic worry (which is, however, distinct from poverty or actual financial distress) as a useful predictor to assess future (after 6 months) depression severity. Specifically, the algorithm showed that students with low or no worries about their economic situation had a greater chance of feeling relatively well with respect to their peers in distress about their finances. There is evidence from previous research (specifically on depressive symptoms and suicidal behaviour) that fear for expected financial losses is sufficient to trigger a cascade leading to depressive symptoms and suicidal thoughts (Fiksenbaum *et al.*, 2017).

However, the association with the largest effect size (i.e., the strongest association) is between suicidal ideation and the severity of depression, with the former increasing the likelihood of severe depression symptoms by about nine times. This has to be contextualized by taking into account that suicidal ideation, measured with BDI, is a measure of depression itself (and a crucial diagnostic criteria for major depressive disorder, as conceptualized by DSM-5 (Cai *et al.*, 2021; Liu *et al.*, 2022)).

Regarding the evaluation of the factors associated with severe symptoms of anxiety, for every one-point increase on the BDI-II somatic subscale, the odds of being severely anxious increase by approximately 9%. In relation to obsessive–compulsive symptoms, we discovered that every extra point on the BDI-II scale increased the chance of developing symptoms by 10%. There could be two interpretations to this finding: the fact that higher scores to the BDI-II tend to increase other scale scores (as a rising tide lifts all boats) and/or both phenomena are comorbid (Jenkins *et al.*, 2021; Norton *et al.*, 2008; Torres *et al.*, 2016). Further studies should consider measuring obsessive, anxiety and depressive symptoms with more than one scale to eventually unravel the contagion effects that a single scale score could exert on the other measures. In other words, in our study, the BDI-II scale was the fastest and useful tool for assessing mental health, but probably for the aforementioned reasons.

When covarying for the depression scores, some variables were significantly associated with the presence of suicidal ideation: we found that the score on the cognitive subscale of depression (which measured the variables related to cognitive distortions) increased the odds of suicidal ideation by 36% for each additional point on the subscale. Marginal significance for mental disorders in the family was evidenced, whereas no history of mental disorders played a significant protective role. It is noteworthy that self-identification with the male gender increased the likelihood of suicidal ideation by 2.84 times. As reported in the literature, depressive symptoms exert a strong effect on suicidal ideation (Konick and Gutierrez, 2005; Wang *et al.*, 2017). Regarding gender, recent evidence contrasted the view that female gender posits a greater risk of suicidal ideation (Eskin *et al.*, 2011; Rogers and Joiner, 2017). The fact that young adult males are at higher risk of suicidal ideation has been getting more traction lastly (Lima *et al.*, 2021; Yarar *et al.*, 2023). These can be better understood in light of the more extensive use of statistical tools, taking into account multiple variables simultaneously and thus examining the corrected weights of gender on suicidal ideation (Gui *et al.*, 2022), although cultural differences need to be thoroughly assessed, as they can shift the weights in favour of males or females (Kaggwa *et al.*, 2023).

Lastly, we employed random forest algorithms to evince what factors at baseline were predictive of a change in depressive symptoms (i.e., still severe symptoms, worsening symptoms, improvement of symptoms and still no severe symptoms) and suicidal ideation (i.e., still suicidal ideation, *ex novo* suicidal ideation, improvement of suicidal ideation or still no suicidal ideation). For both measures, the supervised machine learning algorithm identified the scores on the cognitive and somatic subscales of the BDI, the frequency of economic worry, the field of study and the residence at baseline (the latter only for suicidal ideation) as significant variables to leverage to predict the change (or stability) of depressive symptoms. Noteworthily, suicidal ideation at baseline could be leveraged by the machine to predict suicidal ideation after 6 months, but it did not yield a strictly statistically significant role. To our knowledge, there have been several studies implementing machine learning models leveraging previous suicidal ideation to predict future suicidal ideation (Benjet *et al.*, 2022; Liao *et al.*, 2022; Malone *et al.*, 2021), although time frames from baseline to follow-up did not coincide with our 6-month time frame. Substantially, previous suicidal ideation is an important factor, posing a greater risk of future suicidal ideation and suicidal behaviour (Bafna *et al.*, 2022), but in our analysis, this prediction did not reach canonical statistical significance (although it should be taken into account that the machine learning model would lose a great proportion of its accuracy without data from previous suicidal ideation – Figure 3A). Machine learning in future studies could help find new associations between the variables, although limitations must be considered: when dealing with rare outcomes (e.g., new onset of suicidal ideation after several months from baseline), algorithms learn better to predict the more common outcome (e.g., still no suicidal ideation). We think this unbalance in the data (i.e., outcomes of stability being over-represented with respect to changes in symptomatology) can be effectively dealt with through multicentric collaborations. This approach would simultaneously address two tricky situations: cross-cultural/geographic differences and small sample sizes.

## Strengths and limitations

The main strengths of this study are the prospective design, the analysis of various psychological dimensions through the compilation of validated questionnaires, the large cohort size and the careful acquisition of demographic variables. However, it should be considered that self-reporting questionnaires are not diagnostic instruments, and therefore they might not detect relevant changes in the mental health of participating subjects. Other limits concern the gender distribution of the sample, the response rate and the percentage of follow-up completers. About 1 out of 5 students invited to participate in the study completed all questionnaires. Moreover, approximately 1/3 of the individuals agreed to participate in the study again 6 months after enrolment. Although the baseline differences between follow-up completers and dropouts are negligible in terms of the outcomes of interest, the prospective evidence presented herein must be taken with caution, and it needs further support from more research for the following reasons: a sampling bias could skew the results due to self-selection of the participants, an issue similar to other studies in this field; although the percentage of follow-up completers for online participants is approximately in line with other studies characterized by time-demanding online questionnaires (de Leeuw *et al.*, 2018; Saleh and Bista, 2017), this could be seen as a possible source of participant self-selection. Lastly, it should be mentioned that our sample consists mainly of female participants. It is worth noting that females tend to be more represented than males in the fields of study we sampled. However, the relevant issue of gender bias in willingness to participate in research studies has already been pointed out (Smith, 2008), but the consequences of its effect might be field-specific. In our case, a mixture of skewed gender percentages and self-selection of male participants could have contributed to our finding of the higher likelihood of suicidal ideation in males or, on the other hand, that differences could be due to cross-gender factors that we did not measure. It is worth mentioning that, although valid, we determined suicidal ideation solely on the basis of the BDI-II item 9 response (Desseilles *et al.*, 2012; Green *et al.*, 2015).

## Data Availability

The anonymized dataset and code for analysis can be retrieved at https://www.researchgate.net/profile/Nicola-Meda-2.

## Supplementary material

For supplementary material accompanying this paper visit http://doi.org/10.1017/S2045796023000550. click here to view supplementary material
